# Identification of molecular markers for the *Pc39 gene* conferring resistance to crown rust in oat

**DOI:** 10.1007/s00122-020-03533-z

**Published:** 2020-01-11

**Authors:** Sylwia Sowa, Edyta Paczos-Grzęda

**Affiliations:** Institute of Plant Genetics, Breeding and Biotechnology, University of Life Sciences in Lublin, Lublin, Poland

## Abstract

**Key message:**

Six new PCR-based markers for the *Pc39* crown rust resistance gene in *Avena sativa* L. were developed. *Pc39* was mapped to Mrg11 of the oat consensus map using BLASTn analysis.

**Abstract:**

The aim of this study was the identification of molecular markers for the *Pc39* gene in cultivated oat (*Avena sativa* L.). *Pc39* is a major race-specific crown rust resistance gene originally found in an Israeli accession of the wild hexaploid *Avena sterilis*. The effectiveness of this gene in Europe has decreased in recent years, but is still relatively high and breeding programs would benefit from the availability of molecular markers to aid in its mapping and deployment. The complexity of the oat genome poses a significant obstacle to genetic research. No oat rust resistance genes have yet been cloned, and even the number of relevant molecular markers is very limited. Here, genotyping of a segregating population derived from a cross ‘Celer’ (*Pc39*)/STH9210 (susceptible) was conducted using RAPD- and SRAP-PCR-based methods, as well as microarray-based DArT™ and next-generation sequencing DArTseq™ techniques. Markers associated with *Pc39* were placed on the hexaploid oat consensus linkage group Mrg11 at 3.7–6.7 cM. Six new PCR-based markers were developed to allow identification of the resistant *Pc39* allele. These tightly linked markers will be useful in marker-assisted selection, with the closest, SCAR_3456624, being within 0.37 cM of *Pc39*. The newly developed markers could find applications in the fine mapping or positional cloning of this gene. Moreover, easy-to-use PCR-based markers linked to *Pc39* could facilitate the utilization of this gene in oat breeding programs, especially as a component of crown rust resistance gene pyramids.

**Electronic supplementary material:**

The online version of this article (10.1007/s00122-020-03533-z) contains supplementary material, which is available to authorized users.

## Introduction

Fungal diseases are an important constraint to cereal production. In oats (*Avena* spp.), crown rust caused by *Puccinia coronata* Cda. f. sp. *avenae* P. Syd. & Syd. (Pca) is considered the most destructive fungal disease, causing great yield and grain quality loses (Simons 1985). The chief method of control for crown rust is resistance conditioned by major resistance (R) genes (Cabral et al. 2014). Large-scale cultivation of varieties containing a single R gene increases the probability of emergence of new pathogen races able to overcome this gene. Thus, pyramiding genes with different race specificities may extend their use. Successful gene pyramiding depends upon several factors including efficient identification of component genes (Joshi and Nayak 2010; Kordrostami and Rahimi 2015). So far, selection of rust-resistant oat plants has been based on phenotypic observation using pathogen isolates with defined infection profiles. However, finding the appropriate isolate to detect individual genes is difficult and time-consuming and, in the case of pyramid structures composed of genes with epistatic effects, almost impossible (Kebede et al. 2019). Genotyping and use of markers linked to the genes of interest are a promising alternative to these laborious physiological tests (Tomczyńska and Śliwka 2011; Kordrostami and Rahimi 2015). There are many examples of effective use of marker-assisted pyramiding, such as the introduction of the *Xa21*, *xa5*, *xa4* and *xa13* genes increasing resistance to bacterial blast in rice (Huang et al. 1997; Hittalmani et al. 2000), as well as the *Lr41*, *Lr42*, *Lr43* genes providing resistance to brown rust in wheat (Cox et al. 1993). This method has also proved to be successful in the pyramiding of the *Pm2* + *Pm4a*, *Pm2* + *Pm21*, *Pm4a* + *Pm21* wheat powdery mildew resistance (Liu et al. 2000) or *Rsv1*, *Rsv3*, *Rsv4* resistance to the barley yellow mosaic virus (Werner et al. 2005).

In the *Avena* genus, close to 100 *P. coronata* resistance (*Pc*) genes have been identified (CDL 2018), and much work has been dedicated to chromosome localization and characterization of allelic relationships of these genes (Penner et al. 1993; Rooney et al. 1994; Wilson and McMullen 1997; Bush and Wise 1998; Wight et al. 2004; Kulcheski et al. 2010; McCartney et al. 2011; Gnanesh et al. 2013; Kebede et al. 2019). However, the complexity and size (12.5 GB) of the oat genome present significant obstacles to genetic research, with redundancy being generated by polyploidy as well as the high repetitive DNA content (Jellen et al. 1994; Jellen et al. 1997; Gutierrez-Gonzalez and Garvin 2011; Yan et al. 2016). Cultivated oat (*Avena sativa* L.) is an allohexaploid with basic chromosome number of *x* = 7 and the genome constitution of AACCDD (Rajhathy and Thomas 1974). Due to a high degree of similarity between the A and D genomes, nonhomologous pairing is possible, so the hexaploid oat chromosomes contain substantial rearrangements relative to the basic diploid ancestral chromosomes (Chen and Armstrong 1994; Leggett and Markhand 1995). The presence of numerous multigene families adds a further difficulty to developing linkage maps and assigning map positions to markers (Wight et al. 2003; Gutierrez-Gonzalez and Garvin 2011; Chaffin et al. 2016).

Our previous research has been focused on monitoring of the occurrence and harmfulness of *P. coronata* populations, as well as evaluating the effectiveness and potential of resistance in breeding materials. Results indicate that many *Pc* genes can be still effective against crown rust races occurring in central Europe (Paczos-Grzęda and Sowa 2019); however, the number of molecular markers for those genes useful in breeding is very limited.

*Pc39* is a major race-specific crown rust resistance gene incorporated into common oat from the wild hexaploid oat *Avena sterilis* F-366 collected in Israel and identified by Fleischmann and McKenzie (1968). Šebesta (1983) analyzing virulence of crown rust samples isolated from 1977 to 1980 from various European countries found *Pc39* to be one of the most effective genes at that time. Similar surveys were conducted by Šebesta et al. (1997) from 1990 to 1994, and by Šebesta et al. (2003) from 1995 to 2001, when *Pc39* was also described as highly effective, although *Pc39* virulent isolates were first found. A few years later (2006–2008), Jiráková and Hanzalová (2008) detected no isolates breaking down *Pc39* determined resistance in a set of isolates derived from the Czech crown rust population. The highest frequencies of virulence to *Pc39* in Europe were detected in isolates collected from 2013 to 2015 in Poland, when they ranged from 16 to 31% depending on the year (Paczos-Grzęda and Sowa 2019). Compared to European countries, in the USA, virulence toward *Pc39* analyzed from 2001 to 2005 was very high, especially in *P. coronata* isolates derived from spring rather than winter cultivars (Carson 2011). Similarly, virulence frequencies to *Pc39* isolates from Canada from 2007 to 2009 were over 88% (Chong et al. 2011), even reaching 100% from 2010 to 2015 (Menzies et al. 2019). This means that *Pc39* is still relatively effective in Europe, but wide use of this gene in North American breeding has contributed to the spread of virulence against *Pc39* in *P. coronata* populations in this region.

The only Polish cultivar carrying *Pc39* is ‘Celer.’ This cultivar was selected on the basis of crown rust resistance measurements in a set of new and historical oat cultivars representing 120 years of Polish oat breeding (Sowa and Paczos-Grzęda in press). ‘Celer’ presented the highest level of resistance in seedling and adult plant stages. The infection profile of this cultivar matched the pattern of the reference line for the *Pc39* crown rust resistance gene. Additionally, ‘Celer’ was crossed with the *Pc39* reference line and allelism tests confirmed that the resistance of both is conditioned by the same locus. The cultivar ‘Celer’ is well adapted to central Europe climatic conditions, high yielding and characterized by a number of favorable agronomic traits; therefore, it can be successfully used as a direct *Pc39* gene donor.

The objective of this study was the identification of molecular markers linked to the *Pc39* gene using a F_2_ population derived from a cross ‘Celer’/STH9210, and development of simple, PCR-based markers for this gene, supporting effective *Pc39* gene introduction in breeding programs. Markers closely linked to the gene also facilitated location of the *Pc39* gene on the oat consensus map.

## Materials and methods

### Plant materials

*Pc39* mapping was conducted in F_2_ and F_2:3_ populations derived from a cross ‘Celer’/STH9210 developed at the Institute of Plant Genetics, Breeding and Biotechnology, University of Life Sciences in Lublin, Poland. ‘Celer’ is a spring oat variety produced in the Plant Breeding Station in Borów, Poland with the pedigree Góral//Flämingsnova//Tiger/Pc39//Pan. STH9210 is an oat breeding line developed by the Plant Breeding Company in Strzelce and is susceptible to crown rust infection. PCR markers generated in this project were validated on a set of 22 oat cultivars (Table 4), seven of which are Polish-susceptible oat varieties (Sowa and Paczos-Grzęda in press), and the remaining 15 developed in either AAFC Winnipeg, Manitoba, Canada or North Dakota State University, Fargo, USA, having *Pc39* in their pedigrees.

### Crown rust inoculation

Two hundred twenty seeds of F_2_ ‘Celer’/STH9210 progeny were grown in plug trays filled with a universal substrate containing peat. Two hundred two progeny plants were phenotyped based on the host–pathogen test (Hsam et al. 1997) conducted on the first leaves of 10-day-old seedlings as described in Sowa et al. (2016). Crown rust resistance was tested using three *P. coronata* isolates 13.3/1; 94.1/4 and 107.2/3 (Table 1). The pathotypes were selected from a wide collection of single-pustule isolates derived from populations collected in Poland between the years 2010 and 2014 according to the method described by Sowa et al. (2016). One leaf from each seedling was cut into three 3-cm-long fragments, and fragments were divided across separate 12-well culture plates with agar (0.6%) containing benzimidazole (3.4 mM). A single-leaf fragment of the susceptible cultivar ‘Kasztan’ was used as infection control for each well. Inoculations were performed in a settling tower to provide 500–700 spores of *P. coronata* per 1 cm^2^. Plates were incubated for 10 days in a phytotron at 18 °C with 70% humidity and light intensity of approximately 4 kLx for a 16-h photoperiod.

**Table 1:** Virulence spectrum of *P. coronata* f. sp. *avenae* pathotypes used for testing resistance of the F_2_/F_2:3_ populations derived from ‘Celer’/STH9210

No.	*Puccinia coronata* isolate	Virulence to crown rust differentials
1.	13.3/1	Pc36, Pc38, Pc40, Pc46, Pc55, Pc56, Pc96, Pc97, Pc98, Pc103-1
2.	94.1/4	Pc14, Pc36, Pc40, Pc45, Pc46, Pc51, Pc54, Pc55, Pc57, Pc64, Pc67, Pc70, Pc96, Pc97, Pc98, Pc101, Pc103-1, Pc104
3.	107.2/3	Pc35, Pc38, Pc56, Pc58, Pc67, Pc96, Pc97, Pc103-1

After seedling tests, all individuals were planted in the experimental farm of the University of Life Sciences in Lublin (Czesławice 51° 18′ N, 22° 15′ E). F_2:3_ generation seeds were collected from 155 F_2_ individuals. Sixteen plants from each F_3_ line were tested with *P. coronata* isolates 13.3/1 and 94.1/4 using the host–pathogen methodology cited above.

### Disease rating

Assessment of crown rust disease symptoms was performed after 12 days using 0–4 infection-type (IT) qualitative scale, which was transformed to S, MS, MR, R and HR, where S = 4 = susceptible (large-to-moderately large pustules with little or no chlorosis); MS = 3 = moderately susceptible (moderately large pustules surrounded by extensive chlorosis); MR = 2, 2N, 12C, ;1C = moderately resistant (small pustule surrounded by chlorosis or necrosis); R = ;–N, ;C, ;+ C, 1N = resistant (chlorotic or necrotic flecking); and 0 = HR = highly resistant (no visible reaction) (Murphy 1935; Carson 2009; Sowa et al. 2016) (see Nazareno et al. 2018 for an illustration). Reactions to the crown rust isolate infections were grouped into two classes: Phenotypes described as S and MS were considered as susceptible, and the remainder as resistant.

### DNA extraction

For molecular analysis, genomic DNA was extracted from the frozen tissue of fresh 10-day-old leaf material of all F_2_ individuals using DNeasy Plant Mini Kit (Qiagen). DNA integrity and quality were evaluated by electrophoresis on 1.5% agarose gel. The DNA concentration was determined with NanoDrop2000 spectrophotometry and normalized to 100 ng/μl.

### Bulks preparation

To identify markers linked to *Pc39,* RAPD and SRAP analyses were carried out by BSA (Bulk Segregant Analysis) (Michelmore et al. 1991). Bulks were made by combining equal amounts of DNA from 15 individual homozygous resistant and 15 homozygous susceptible F_2_ plants. Homozygosity was determined by inoculation of F_3_ families with crown rust isolates 13.3/1 and 94.1/4 as above.

Screening of RAPD and SRAP primers was performed by comparing the amplification products from bulks of DNAs from resistant and susceptible plants as well as DNAs from population parental forms and DNA from line Pc39, the initial source of the *Pc39* crown rust resistance gene.

### RAPD analysis

PCR reactions were performed according to the RAPD method described by Williams et al. (1990) with modifications. The 520 primers used correspond to Operon Technologies kits A–Z (https://www.eurofinsgenomics.eu). Amplifications were carried out in final volumes of 10 µL containing 20 ng of template DNA, 1 × DreamTaq™ Buffer (750 mM Tris–HCl (pH 8.8 at 25 °C), 200 mM (NH_4_)_2_SO_4_, 0.1% (v/v) Tween 20) with 2 mM MgCl_2_, 0.5 U DreamTaq™ DNA polymerase (Thermo Fisher Scientific), 0.16 mM of each dNTP and 0.8 µM oligonucleotide primer. The PCR was completed using the Biometra T1 Professional Basic thermocycler programmed for 3 min in 95 °C of initial denaturation, 45 cycles: 94 °C–45 s, 37 °C–45 s, 72 °C–1 min, with a final extension at 72 °C for 7 min.

### SRAP analysis

For the SRAP assay, the PCR mix of 10 µL final volume contained 25 ng of template DNA, 1 × DreamTaq™ Buffer, 2 mM MgCl_2_, 0.6 U DreamTaq™ DNA polymerase (Thermo Fisher Scientific), 0.2 mM of each dNTP and 0.4 µM of forward and reverse primers. The amplification protocol was as follows: 94 °C for 3 min (pre-denaturation); five cycles of 94 °C for 1 min, 35 °C for 1 min, and 72 °C for 1 min; 36 cycles of 94 °C for 1 min, 50 °C for 1 min, and 72 °C for 1 min; and a final extension of 72 °C for 7 min (Li and Quiros 2001). Thirty-two forward and 32 reverse SRAP primers were used in 1032 primer combinations. The core forward (5′-TGAGTCCAAACCGG-3′) and reverse (5′-GACACCGTACGAATT-3′) primer sequence was followed by three selective, but random nucleotides (Li and Quiros 2001; Budak et al. 2004).

### PCR products separation

Products of amplification were separated on 1.5% agarose gel containing 5 μg/ml EtBr in 1xTBE Buffer (90 mM Tris–borate, 2 mM EDTA, pH 8.0). To establish molecular weight of the products, Gene Ruler™ 100 bp Plus DNA Ladder was used. Fragments were visualized under UV transilluminator and photographed.

### DArT and DArTseq™ analysis

The microarray-based DArT and DArTseq™ assay were performed at Diversity Arrays Technology Pty Ltd. (DArT P/L), Canberra, Australia. Development of DArT markers followed methods published by Wenzl et al. (2004) and Tinker et al. (2009) and is described in detail by Kilian et al. (2012). DArTseq™ technology combining DArT with a next-generation sequencing technique was performed as in Courtois et al. (2013). DArTseq™ provided two types of markers: silicoDArT presence/absence variants (PAVs) and DArTseq single-nucleotide polymorphisms (SNPs).

### SCAR primer design and marker validation

RAPD-PCR and SRAP-PCR products which differentiated the DNA pools were verified by tests on 20 random homozygous individuals, then extracted from agarose gels and purified using the GenElute™ Gel Extraction Kit (Merck). Purified DNA was cloned using the TOPO^®^ TA Cloning^®^ Kit for Sequencing (Invitrogen) and competent cells of *E. coli* strain DH5α™-T1^R^. Plasmids were extracted from bacterial cells and used as a template for PCR with M13 forward/reverse primers. Products were subjected to Sanger sequencing at Genomed (Warsaw, Poland). Sequences were analyzed using BioEdit sequence alignment editor v. 7.0.5.3 (Hall 1999) and used to design the sequence-characterized amplified region (SCAR) primers with NCBI primer blast (Ye et al. 2012) as well as Primer3 software (Rozen and Skaletsky 2000).

DArT, silicoDArT and DArTseq markers with segregation patterns closest to the crown rust resistance phenotype in the studied population (Spearman’s rank correlation *r* < 0.8; *p* < 0.001 (Spearman 1904)) were chosen for further analysis. DNA sequences were analyzed to identify primer pairs for their amplification as above. The criteria for primer design were as follows: 40–60% GC rich; minimum annealing temperature, 50 °C; and no or negligible secondary structures.

SCAR PCR was carried out in 10-μl reactions. For RAPD-SCAR, DArT-SCAR and silicoDArT-SCAR, reactions contained 20 ng of template DNA, 1 × Taq PCR Buffer (750 mM Tris–HCl, pH 8.8 at 25 °C; 500 mM KCl; 0.8% v/v Nonidet P-40; 1,25 mM MgCl_2_), 0.5 U Taq DNA polymerase, (Thermo Fisher Scientific), 0.1 mM of each dNTP and 0.35 µM of each forward and reverse oligonucleotide primer. The PCR thermal profile consisted of an initial hold at 94 °C for 3 min, followed by 38 cycles of 94 °C for 25 s, 58 °C for 30 s and 72 °C for 1 min 10 s, followed by a final hold at 72 °C for 5 min. For SRAP-SCAR, reactions contained 20 ng of template DNA, 1 × Taq PCR Buffer 2 mM MgCl_2_, 0.6 U DNA polymerase (DreamTaq™, Thermo Scientific), 0.2 mM of each dNTP and 0.4 µM of forward and reverse primers. For DArTseq, the polymerase was changed to JumpStart™ Taq and the buffer was changed to JumpStart™ (Merck). Thermocycling conditions were as follows: an initial denaturation at 94 °C for 5 min, 38 cycles of 94 °C for 30 s, 49 °C for 30 s and 72 °C for 30 s, ended with final extension at 72 °C for 5 min. PCR products were visualized on agarose gels as described above.

The predictive ability for obtained PCR markers linked to the *Pc39* gene was evaluated using a set of 22 oat cultivars (Table 4).

### Statistics

Chi-squared (*χ*^2^) analyses of the phenotyping data from F_2_ and F_3_ progeny were conducted in order to test the goodness-of-fit of observed to expected segregation ratios.

### Sequence data analysis

Sequence homology searches were performed with the BLASTn at http://www.ncbi.nlm.nih.gov of the National Center for Biotechnology Information (NCBI) using the Nucleotide Collection Database. The threshold parameter was established at 10^−7^, with only hits with *E* values below this cutoff considered as significant. BLASTn with basic search options was also performed at the T3/Oat web interface (https://triticeaetoolbox.org/oat/viroblast/viroblast.php) to assign positions of selected markers to the consensus map of Chaffin et al. (2016) and Bekele et al. (2018).

### Linkage analysis

MapDisto 2.0 software (Lorieux 2012) was used to create a partial linkage group from the DNA marker data. Loci were placed into linkage groups using a minimum LOD (logarithm of odds) score of 3 and a maximum distance between markers of 30 cM. Individuals with > 20% of missing data were omitted. Marker order was determined with the Seriation II method based on the Seriation algorithm (Buetow and Chakravarti 1987) using the SARF (Sum of Adjacent Recombination Frequencies) criterion. Graphical linkage group presentation was generated using MapChart 2.32 Software (Voorrips 2002). Linkage groups were assigned to the oat consensus map of Chaffin et al. (2016) and Bekele et al. (2018).

## Results

### Segregation of resistance to crown rust in the ‘Celer’/STH9210 F_2_ and F_2:3_ populations

The *P. coronata* isolates used to assess segregation of *Pc39* resistance gene were virulent to the STH9210 line and were avirulent on ‘Celer.’ Based on Chi-square tests, the segregation patterns fit a Mendelian ratio of three resistant/one susceptible genotype in F_2_ population and one homozygous resistant/two segregating/one homozygous susceptible genotypes in F_3_ population confirming a single gene hypothesis. The detailed results were 145 R: 35 S with the 13.3/1 *P. coronata* isolate (*χ*^2^ = 2.96; *p* value = 0.085); 144 R:34 S with 94.3/1 *P. coronata* isolate (*χ*^2^ = 3.02; *p* value = 0.083) and 144 R:33S with 107.2/3 *P. coronata* isolate (*χ*^2^ = 3.66; *p* value = 0.056). The isolates 13.3/1 and 94.1/4 used to screen 155 F_2:3_ families both generated results of 49 R/70 H/36 S (*χ*^2^ = 3.43; *p* value = 0.179) (Table 2).

**Table 2:** Segregation ratios of F_2_ progeny and F_2:3_ families from the cross ‘Celer’/STH9210 when inoculated with crown rust isolates 13.3/1; 94.1/4 and 107.2/3

‘Celer’/STH9210							
Generation	*Puccinia coronata* isolate	Resistant	Segregating	Susceptible	Ratio	*χ*^2^	*p* value
F_2_	13.3/1	145	–	35	3:1	2.963	0.08519*
F_2:3_	13.3/1	49	70	36	1:2:1	3.431	0.17985*
F_2_	94.1/4	144	–	34	3:1	3.019	0.08229*
F_2:3_	94.1/4	49	70	36	1:2:1	3.431	0.17985*
F_2_	107.2/3	144	–	33	3:1	3.659	0.05574*

*Nonsignificant

### Bulked segregant analysis

The 520 RAPD primers tested amplified a total of 5420 DNA products. Nineteen of these potentially differentiated resistant and susceptible hybrid pools in the ‘Celer’/STH9210 population. One-thousand twenty-four SRAP primers tested amplified 12,325 products, with 35 selected as generating polymorphic bands between the contrasting DNA bulks. Only two combinations (OPH11; Me23 + Em14) were validated as amplifying products which matched the presence or absence of the *Pc39* gene in 20 random individual plants. The primer OPH11 5′-CTTCCGCAGT-3′ generated a ~ 1100 bp DNA fragment which was present in the susceptible bulk and individuals as well as the STH9210 susceptible parent. SRAP primers Me23: 5′-TGAGTCCAAACCGGGAT-3′ and Em14: 5′-GACTGCGTACGAATTCTA-3′ generated a ~ 900 bp DNA fragment present in the resistant bulk and individuals, Pc39 line and resistant parent ‘Celer.’ The PCR products were cloned and sequenced.

### Identification of DArT, DArTseq and silicoDArT markers correlated with *Pc39* segregation pattern

Six hundred ten DArT, 29430 DArTseq and 3600 silicoDArT markers had been generated, 15 of which (1 DArT and 14 silicoDArT) were highly correlated with the *Pc39* dominant allele segregation pattern, whereas thirty-nine (3 DArTseq and 36 silicoDArT) corresponded to the *Pc39* recessive allele segregation pattern (Supplemental Table 1).

### Sequence homology analysis

Significant BLASTn hits at NCBI database were obtained for the SCAR_SRAP_Me23 + Em14 (907 nt). The sequence matched five regions of *Avena strigosa* accession DQ680849.1 containing beta-amyrin synthase (*Sad1*) and cytochrome P450 CYP51H10 (*Sad2*) genes sequences (*E* value of 1e−165, 92% query coverage) and showed a significant similarity to the *Pc68LrkB1* (AY038003; three regions; *E* value of 2e−137, 92% query coverage), *Pc68LrkB6* (AY038008; three regions; *E* value of 0, 92% query coverage), *Pc68LrkC1* (AY038009; two regions, *E* value of 5e−158, 92% query coverage) and *Pc68LrkC3* (AY038011; two regions (*E* value of 2e−69, 53% query coverage). These sequences are four subclones generated by sequencing of Pc68LrkB (12,467 bp) and Pc68LrkC (11,747 bp) clones isolated from the Pc68 line. The sequences contain retrotransposon and repetitive DNA linked to the ALrk receptor kinase gene.

**Fig. 1:**
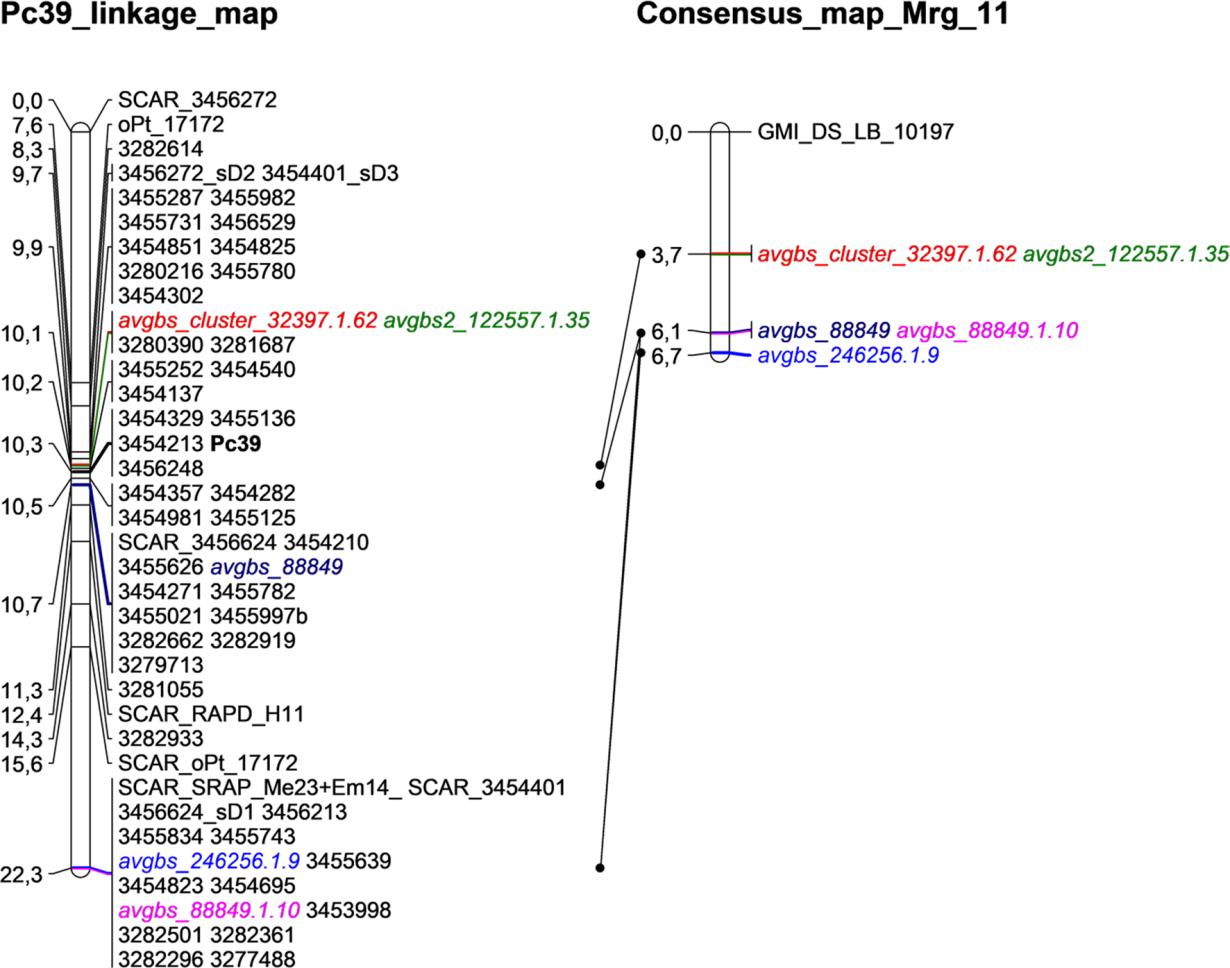
Partial linkage map of markers for *Pc39* gene and its assignment to Mgr11 group of the oat consensus map developed by Chaffin et al. (2016) and saturated by Bekele et al. (2018). Pc39 linkage map contains DArT (oPt_), DArTseq (marked with numbers) and silicoDArT (_sD) markers for Pc39 gene as well as SCAR (sequence-characterized amplified region) markers based on DArT (SCAR_oPt_), DArTseq (SCAR_), RAPD-PCR (SCAR_RAPD), SRAP-PCR (SCAR_SRAP) products correlated with *Pc39* segregation pattern

BLASTn at the T3/Oat web revealed that silicoDArT 3455235 was highly similar to avgbs_cluster_32397.1.62, and 3,456,198 was homologous to avgbs2_122557.1.35. Both of those markers are localized on the position 3.7 of Mrg11 of oat consensus map. Additionally, 3455179 and 3455725 were highly similar to avgbs_246256.1.9 of Mrg 11 (6.7). SilicoDarT 3454180 was also highly similar to avgbs_88849.1.10 (Mrg11_6,1) (Supplemental Table 1; Fig. 1).

### SCAR marker design

In order to increase the specificity and reproducibility of RAPD and SRAP markers, a set of SCAR primers was designed based on the sequenced DNA fragments (Table 3). Amplifications were carried out for the parents and the full set of ‘Celer’/STH9210 F_2_ progeny. With SCAR_RAPD_H11 primers (SCAR_RAPD_H11 _F 5′-CTTCCGCAGTCTTACCTATTT-3′; SCAR_RAPD_H11_R 5′-CTTCCGCAGTGGTGTGGT-3′), a single specific band of 1111 bp was amplified in susceptible parent STH9210 and most of the susceptible and heterozygous plants. The background DNA fragments amplified by the decamer primer OPH11 were no longer detected on the gel. With SCAR_SRAP primers (SCAR_SRAP_Me23 5′-ATGCTCGTCCCCTATCTTCA-3′, SCAR_SRAP_Em14 5′-CGTACGAATTCTTTAC-3′), a DNA product of 707 bp matching the segregation of the *Pc39* dominant allele was amplified.

**Table 3:** DNA sequences used for SCAR makers design

Sequence name	Sequence (5′–3′)	Annealing temp. (°C)
SCAR_RAPD_H11	**CTTCCGCAGTCTTACCTATTT**TGTAGTCACATTTATTTCGTTTGGAAAAGCTACCTCTACCATGTGCACCAAGTACTGCACCTACTATGCCTGTTTCTTTCGGCATGTAAAAGAACAACTCAGGTAAGTACGTACGTACTGTACGTGCTAGCTGAAGATGATCGTGGTTGTGTGCGTCGCCATGATATCCTCGGTCTCGTCTTCACCGGGTGCGTAGTCTTTGCGCTTGTATACTTTCACCACGAGTTGCTAGGGAGTTGCCGAACACGCCCTACGTTCTAATTGCCCTCCCAAGCTATCACCCTCCTTCCTTCCTGAGACAGAATCTTTAATCGACACCTAAAAATCTTTAATTGGCCAGATGAATAGTACATCTTAAACAAAGTCTACACACATTATCTCTCTACCTTTTTATTTTGAAAAGAATATGGATGTCCCTATCACCGATCTATTTTTAAGCTAACTAGCAGCGACTCGGTGCACTGAATACCAACGAAGCCAAGAGCGAGGAGTACGTAGGAAACGCACCGCCTACGATGCGTGTTGTCCTAGCAAACCGATAAGTAATGGAACATGTGCCGTTTATAACAGTCATAGTACTATGTACAATAGTTTACACGTATACATTAACATTATCTTCACATATGGAACTCAGTTGACACCTAGCTAAAATTCTTGAATTTGTCAGATGAATAGTTACATCTTAAACAAAGTCTACACGCAATATCTGCCTACCTTTTATTTTAAAAAAGAATATTGTCATTGATGTTACACTCATGCCCCCATCACCGATCTATCTTTATTTTGAGAAAGAATATATTGTCATATCGACTCGATGCACTGAATTCCAACCAAGCTGCCAAGACCAAGAGTGAGGAGTAGGATGCACCACGTATGTACGATAAGTAATAAAATACGTGCCATTTATAATAATATTCGTAGATTACACGTGTACATTAACATTATCTTCATGTTTGGAACTCGGTACTGCTAGTCAATCGTAGCCTAATAAATTAGGCAGAGGTTGCTTGTTTGACCTGATCGCATCCACTCTCTATAAAATACGGCACCCTACTGATGCCKCCTTGTACAA**ACCACACCACTGCGGAAG**	58
**SCAR_SRAP**	TTATGTYCAAACKCGGGATATCTCAAAACATTTCSGTMCATSCCGGGGTAAAAAAGGACAAAATGTCTGCCGAATCGGTAGGAAGTGGGTCCGGTTTGTACTACAGGTACATGATCTAACGCCCGTGATTTTTTGAAAAAAAACATTTTTAGACTCACAATATGTGGTTTCTTCAGAGATCCAATGCAAGTTCTAATG**ATGCTCGTCCCCTATCTTCA**TGCACGCCGGTTCGACCTTAGTTCGAAAAGGGAAAAATTCAAAGCAGGCATAAGATATTCAAAAAAGTTCGAACAATGTAAATCCTTCGTGTGGTGTCATATTATGTGACATAGTTGCAAGGAAAAATGCCAGACTTGGTATATTGATCATCTCTCGAAAAAACCTTCACAAAATGACCTGTCATGTTCGAGGTTTCATGGCTTTCGGTCAAATGACCAATGTTATGTGCTGAATCGCGGCATAGTTTATGATATTGTGCCCATATTATGCATGTATGTGGAAATTGGGATGGAGCACAATGTTGCAGGAGGAAGTTTTCCTTTTCGTTACACGGAAAAGCCATTTTCCATTTTCCGAGCGCAAAAAACGGGTCGTTTTGTAAAGCAACCACCAAAATGCTGTTTCAGAATGCTACCGTCCCATTTTTCTAAAATACTAGACCACCTTACATGGACTATCCTCCAACCGGGATATCTCAAACTTTTCCGTCCATCCCAGGTAAAAAGGATAAATTTCTACCGAATCAGTAGGAAGTAGGGTTTGTACTACAGATACATGATCTAACGCCCGTGATTTTTTGAAAAAATCATTTTTAGACTCGCAATATGTGGATTCTTTAGAGATCCAATGCAAGTTCTAAATAAGAAATAAGCCCATTTAGTCCCGGTTG**GTAAAGAATTCGTACGC**A	52
**oPt-17172**	TGCAGGCACAACCGACATTCCTATGCATCCTTTGTCCCATCAGGAAACGGTCCATCTATGTTCACCCTGACAAAACCGGCAGGAGGACGAGTACATTTT**GAAACGTGGGTTATGCTCGG**CGCACTCTTCAGGGTGCAGAGTTCTTTCTATTCAGCGACATATCTTGTAATCTGATACAGAAATTCACCAACTCCTGTAAGAGACTCAACATGGTTTGCCTTATTGTGCTGCACCCAAAACAGAGGAAACAGAGTGAGCGCTACTGTAGCTCATCCGACAATGCGAATATGCTCGCCAAAATTCTCATTGCACAATAAAAGTTCCTGACGGATACCCGTCCGTTAAGCTGCTAACCACGTCTTCTTCACCTCATTGCACATCATGCCCAAACTGTACTGACTGTAATGATGCGTAATAGTCAGACGTTCGGTAGAACTCTTAATCCTGATAGATATTACAGTCACAGAGCAGGTATAGCTATTTACAGATGGGTTCCCGG**TTAGAGAGTACGCGGCGTCT**GCA	65
**3456624**	**TGCAGTGAGCTAGTTTAGTT**CATCTGCGTCGTCGTTAGAG**ACTCTGGATTCATGCTTGAA**CGACGTAGT	58
**3456272**	**TGCAGCAGGTATGGAAGTGAC**GT**CGCACCGCGCTGTCAATTC**AGCCGAGATCGGAAGAGCGGTTCAGCA	65
**3454401**	TG**CAGAGGAGTTGCTACTAGTACATGG**AAATGAATCTCCTCTG**CTTGGCACTTCTTCCCGTGA**AGGCCC	56

The bold, underlined regions are the sequences used for SCAR marker design. Annealing temperature for each primer set is given in the last column

For DArT, DArTseq and silicoDArT markers meeting the criteria for primer design, pairs of SCAR primers were synthesized (Table 3). DArT SCAR_oPt_17172 marker co-segregated with the recessive susceptible *Pc39* allele and silicoDArT SCAR_3456624, SCAR_3456272 and SCAR_3454401 co-segregated with the dominant resistant *Pc39* allele.

### Linkage analysis and marker validation

SCAR, DArT, DArTseq and silicoDArT markers linked to the *Pc39* gene were used to create a partial linkage group (Fig. 1). Six PCR-based dominant markers were mapped from 0.37 cM (SCAR_3456624) through 2.2 cM (SCAR_RAPD_H11), 5.3 cM (SCAR_oPt_17172) to 12 cM (SCAR_SRAP_Me23 + Em14; SCAR_3454401) from *Pc39* (Fig. 1).

The best diagnostic marker for *Pc39* was SCAR_3456624, identifying all tested oat cultivars which had a *Pc39* gene donor in their pedigree (Table 4).

**Table 4:** Evaluation of the predictive ability of markers linked to the *Pc39* gene using a set of oat cultivars varying for the presence of *Pc39*

Oat cultivar	Plant identifier	Pedigree	Potential *Pc* resistance genes present in a cultivar	Origin	SCAR_3456624 (60 bp)	SCAR_3456272 (42 bp)	SCAR_3454401 (61 bp)	SCAR_SRAP Me23 + Em14 (707 bp)	SCAR_RAPD_H11 (1111 bp)	SCAR_oPt_17172 (420 bp)
‘Celer’	–	‘Góral’/KR-KOR	*Pc39*	Plant Breeding Station Borów, Poland	A	A	A	A	–	–
STH9210	–	(STH212/STH13817)/(STH214/STH13827)	–	Plant Breeding Strzelce, Poland	–	–	–	–	B	B
AC ‘Assiniboia’	CN 106513	‘Robert’/90GC144	*Pc38, Pc39, Pc68*	AAFC Winnipeg, Manitoba, Canada	A	–	–	–	B	–
AC ‘Medallion’	CN 106509	90GC142,143/’Dumont’	*Pc38, Pc39, Pc68*	AAFC Winnipeg, Manitoba, Canada	A	A	A	–	–	–
AC ‘Pinnacle’	CN 99035	91RAT20/AC ‘Medallion’	*Pc38, Pc39*, *Pc68*	AAFC Winnipeg, Manitoba, Canada	A	A	A	–	–	–
AC ‘Rebel’	CN 99043	‘Valley’/OT252	*Pc38*, *Pc39*	AAFC Winnipeg, Manitoba, Canada	A	A	A	A	–	B
‘Dumont’	CN 42932	‘Harmon’ HAM/Double Cross 7	*Pc38, Pc39*	AAFC Winnipeg, Manitoba, Canada	A	A	A	–	–	–
AC ‘Ronald’	CN 99039	W89329 (dwarf)/AC ‘Medallion’	*Pc38, Pc39, Pc68*	AAFC Winnipeg, Manitoba, Canada	A	–	–	–	B	–
‘Furlong’	CN 113598	W93069 (Pg16)/AC ‘Assiniboia’	*Pc38, Pc39, Pc68*	AAFC Winnipeg, Manitoba, Canada	A	–	–	–	B	–
‘Jordan’	CN 113506	OT377/’Ronald’	*Pc38, Pc39, Pc68*	AAFC Winnipeg, Manitoba, Canada	A	A	A	A	B	–
‘Riel’	CN 17835	RL3057/’Otana’	*Pc38*, *Pc39*	AAFC Winnipeg, Manitoba, Canada	A	A	A	–	–	–
‘Robert’	CN 99034	OT212/Double Cross 7	*Pc38, Pc39*	AAFC Winnipeg, Manitoba, Canada	A	–	–	–	B	–
‘Stainless’	CN 113504	unnamed_14823/’HiFi’	*Pc38, Pc39, Pc68, Pc91*	AAFC Winnipeg, Manitoba, Canada	A	–	–	A	–	–
‘Steele’	CN 43115	unnamed_6671/’Noble’	*Pc38, Pc39*	AAFC Winnipeg, Manitoba, Canada	A	A	A	–	–	–
‘Summit’	CN 113503	‘Ronald’/OT299	*Pc38, Pc39, Pc68*	AAFC Winnipeg, Manitoba, Canada	A	–	–	–	B	–
‘Newdak’	PI 540399	unnamed_6609/’Ogle’	*Pc38, Pc39*	North Dakota State University, Fargo, USA	A	A	A	–	–	–
‘Valley’	PI 525183	unnamed_2588/’Porter’	*Pc38, Pc39*	North Dakota State University, Fargo, USA	A	A	A	–	–	–
‘Bingo’	–	STH 214/STH 13827	–	Plant Breeding Strzelce, Poland	–	–	–	–	B	B
‘Borys’	–	‘Dato’/Po.39//’Pinto’	–	Plant Breeding Borów, Poland	–	–	–	–	–	–
‘Dragon’	–	MGH 6374/’Diadem’	–	Plant Breeding Rogaczewo, Poland	–	–	–	–	–	–
‘Dukat’	–	‘Fagot’ × KR 2335/74	–	Plant Breeding Strzelce, Poland	–	–	–	–	B	B
‘Farys’	–	‘Biały Mazur’/’Astor’//Cebeco 7511	–	Plant Breeding Polanowice, Poland	–	–	–	–	–	–
‘Góral’	–	‘Borek’/’Brutus’	–	Plant Breeding Strzelce, Poland	–	–	–	–	–	–
‘Karol’	–	STH 171/’Brutus’	–	Plant Breeding Strzelce, Poland	–	–	–	–	B	B

A, maternal allele carrier; B, paternal allele carrier

## Discussion

DNA markers are a key element of modern genomics and a useful tool for resistance breeding, enabling fast and reliable gene identification at early stages of plant development. In this research to develop markers for *Pc39*, different molecular marker systems based on distinct polymorphism-detecting techniques were used.

The only marker for *Pc39* identified to date was a RFLP marker cdo666 found in the Pendek3948 population and located 6 cM away from the gene (Wight et al. 2004). This marker was present in Pendek3948 linkage groups 1 and 5. Group 1 shows homeology to KO37. Group 5 is homeologous to group 3, and both these groups show homeology to KO group 16_23 of the Kanota/Ogle genetic map (Wight et al. 2003), based on the single marker cdo1090d. RFLP marker systems require a complicated and costly procedure (Tanksley et al. 1989), which precludes high-throughput application. In this study, we have focused on the development of PCR-based markers for *Pc39*, which are simple to assay, requiring only a thermocycler and agarose gel electrophoresis. Markers linked with the *Pc39* gene were successfully used for the development of six new PCR-based screens suitable for breeder selections. It is unlikely that one marker will be applicable across all tested germplasm, unless it directly differentiates alleles defining the trait, so the availability of multiple independent markers is desired. Use of the SCAR_RAPD_H11_ F/R and oPt-17172_F/R primers allows the identification of the susceptible *Pc39* allele, while primers SCAR_SRAP_Me23/Em14, 3456624_F/R, 3456272_F/R and 3454401_F/R can be used to identify the resistant *Pc39* allele. All developed markers are dominant so that simultaneous detection of both alleles in a single reaction using multiplex PCR will allow selection of heterozygote genotypes (Elnifro et al. 2000; Hayden et al. 2008).

The availability of the consensus map of Chaffin et al. (2016) and the subsequent version of Bekele et al. (2018) significantly simplified gene mapping to linkage groups. Genome-wide association mapping of crown rust resistance in elite oat germplasm (Klos et al. 2017) confirmed previously determined locations of *Pc38* (Mrg02 chromosome 9D) and *Pc48* (Mrg20) (Wight et al. 2004) as well as *Pc58a* (Mrg02; Hoffman et al. 2006; Jackson et al. 2007), *Pc68* (Mrg19; Kulcheski et al. 2010), *Pc71* (Mrg21; Bush and Wise 1998) and *Pc91* (translocated chromosome 7C-17A, Mrg18, Mrg28; Gnanesh et al. 2013). Continuous progress in chromosome localization of *Pc* genes enables the development of genomic tools in oat breeding but also facilitates the characterization of allelic relationships of these genes. Because of the highly dynamic nature of *P. coronata* populations, race-specific crown rust resistance can be easily overcome, creating an urgent need to identify new effective sources of resistance. Confirmation that sources do represent novel variation is difficult without information about the genomic location of existing *Pc* genes. For example, a seedling resistance gene identified in the oat cultivars Kame and Morton (McMullen et al. 2005; Gnanesh et al. 2015) was described as a locus, *PcKM,* and was placed on Mrg08 between 80.2 and 81.4 cM (chromosome 12D). Recent studies of Kebede et al. (2019) showed that *Pc45* has the same location as *PcKM*, suggesting that these are the same genes. Moreover, Admassu-Yimer et al. (2018), thanks to expanded consensus map data availability, were also able to place *Pc53* in close proximity to this gene (Mrg08, 82.4 cM), suggesting that *Pc53* and *Pc45* may be components of a gene complex in this region conditioning resistance to *P. coronata*.

Many *Pc* genes presumably occur in clusters (Harder et al. 1980; Martens et al. 1980; Chong et al. 1994; Chong and Brown 1996; Leonard et al. 2005). Development of molecular markers for one of the *Pc* genes could determine its location in the genome and may facilitate the study of other genes present in the same cluster or may allow it to be distinguished from flanking loci. According to Kiehn et al. (1976) *Pc39* gene is clustered with *Pc55*, while Leonard et al. (2005) hypothesize that *Pc39*, *Pc55* and *Pc71* are alleles of the same gene. Wight et al. (2004) suggest that *Pc39* may be present on KO group 16_23, which according to Chaffin et al. (2016) is homeologous to Mrg24 of consensus map of oat or KO37 equivalent to Mrg11. In this study, we used all identified marker sequences co-segregating with *Pc39* to perform BLASTn at the T3/Oat web interface to assign the approximate *Pc39* positions on the consensus map of oat. Our data showed that *Pc39* is linked with markers placed on the *Avena* consensus linkage group Mrg11 at 3.7 to 6.7 cM. The closest marker, SCAR_3456624, is also the best diagnostic marker, allowing the identification of the *Pc39* dominant allele in all tested oat cultivars having *Pc39* in their pedigree. Linkage with crown rust resistance has not been previously reported for this genomic region; however, Klos et al. (2017) detected a QTL for crown rust reaction on Mrg11 at 16.2 to 21.9 cM in the spring oat panel. This experiment used a set of eight isolates virulent against *Pc39*, so the identified region could not be associated with the *Pc39* gene. According to Bush and Wise (1998), *Pc71* is located on Mrg21 which excludes the possibility that *Pc39* and *Pc71* are alleles of the same gene.

BLASTn against the NCBI database did not show the significant sequence similarity for any of the analyzed markers, which may be due to the lack of the sequence available for the *Avena sativa* genome, which in turn significantly limits the ability to identify candidate genes for *Pc39*.

Expanding knowledge of the genetics of host resistance is a key to resistant cultivar development. The large and complex oat genome structure, with its high proportion of repetitive elements, pseudogenes, low gene density and polyploidy, poses a serious challenge to oat improvement. The newly developed PCR-based markers for *Pc39* are closely linked to the gene and bracket the locus. They will support effective gene introgression and facilitate fine mapping and positional cloning. Such study may also contribute toward better understanding of crown rust resistance mechanisms in oat and provide insight into genes function, as none of the *Pc* genes has been cloned to data.

## Electronic supplementary material

Below is the link to the electronic supplementary material.

Supplementary material 1 (XLSX 14 kb)
